# Genetic mutation patterns among glioblastoma patients in the Taiwanese population – insights from a single institution retrospective study

**DOI:** 10.1038/s41417-024-00746-y

**Published:** 2024-02-28

**Authors:** Yu-Fen Huang, Ming-Tsang Chiao, Tzu-Hung Hsiao, Yong-Xiang Zhan, Tse-Yu Chen, Chung-Hsin Lee, Szu-Yuan Liu, Chih-Hsiang Liao, Wen-Yu Cheng, Chun-Ming Yen, Chih-Ming Lai, Jun-Peng Chen, Chiung-Chyi Shen, Meng-Yin Yang

**Affiliations:** 1 Department of Neurosurgery, Neurological Institute, Taichung Veterans General Hospital, Taichung, Taiwan; 2 Department of Medical Research, Taichung Veterans General Hospital, Taichung, 40705 Taiwan; 3 Precision Medicine Center, Taichung Veterans General Hospital, Taichung, 40705 Taiwan; 4 Doctoral Program in Translational Medicine, National Chung Hsing University, Taichung, Taiwan; 5 Rong Hsing Translational Medicine Research Center, National Chung Hsing University, Taichung, Taiwan; 6 Institute of Medicine, Chung Shan Medical University, Taichung, 402 Taiwan; 7 Graduate Institute of Life Science, Department of Life Science, College of Life Science, National Chung Hsing University, Taichung, Taiwan; 8 Department of Post-Baccalaureate Medicine, College of Medicine, National Chung Hsing University, Taichung, Taiwan; 9 School of Medicine, Taipei Medical University, Taipei, Taiwan; 10 Department of Minimally Invasive Skull Base Neurosurgery, Neurological Institute, Taichung Veterans General Hospital, Taichung, Taiwan; 11 Department of Physical Therapy, Hung Kuang University, Taichung, Taiwan; 12 Institute of Biomedical Sciences, National Chung Hsing University, Taichung, Taiwan; 13 School of Medicine, National Defense Medical Center, Taipei, Taiwan; 14 Department of Critical Care Medicine, Taichung Veterans General Hospital, Taichung, Taiwan; 15 Functional Neurosurgery Division, Neurological Institute, Taichung Veterans General Hospital, Taichung, Taiwan; 16 Institute of Molecular Biology College of Life Science, National Chung Hsing University, Taichung, Taiwan; 17 Biostatistics Task Force, Taichung Veterans General Hospital, Taichung, Taiwan; 18 Basic Medical Education Center, Central Taiwan University of Science and Technology, Taichung, Taiwan

**Keywords:** Cancer genetics, CNS cancer

## Abstract

This study utilized Next-Generation Sequencing (NGS) to explore genetic determinants of survival duration in Glioblastoma Multiforme (GBM) patients. We categorized 30 primary GBM patients into two groups based on their survival periods: extended survival (over two years, *N* = 17) and abbreviated survival (under two years, *N* = 13). For identifying pathogenic or likely pathogenic variants, we leveraged the ClinVar database. The cohort, aged 23 to 66 (median: 53), included 17 patients in Group A (survival >2 years, 10 males, 7 females), and 13 patients in Group B (survival <2 years, 8 males, 5 females), with a 60% to 40% male-to-female ratio. Identified mutations included *CHEK2* (c.1477 G > A, p.E493K), *IDH1* (c.395 G > A, p.R132H), and *TP53* mutations. Non-coding regions exhibited variants in the *TERT* promoter (c.-146C > T, c.-124C > T) and *TP53* RNA splicing site (c.376-2 A > C, c.376-2 A > G). While Group A had more mutations, statistical significance wasn’t reached, likely due to sample size. Notably, *TP53*, and *ATR* displayed a trend toward significance. Surprisingly, *TP53* mutations were more prevalent in Group A, contradicting Western findings on poorer GBM prognosis. In Taiwanese GBM patients, bevacizumab usage is linked to improved survival rates, affirming its safety and effectiveness. *EGFR* mutations are infrequent, suggesting potential distinctions in carcinogenic pathways. Further research on *EGFR* mutations and amplifications is essential for refining therapeutic approaches. *TP53* mutations are associated with enhanced survival, but their functional implications necessitate detailed exploration. This study pioneers genetic analysis in Taiwanese GBM patients using NGS, advancing our understanding of their genetic landscape.

## Introduction

Glioblastoma multiforme (GBM), a notoriously aggressive primary brain tumor, poses significant challenges in management and treatment due to its rapid progression and dismal prognosis. Over the years, intensive research has sought to unravel its complex pathogenesis, focusing on genetic mutations and exploring novel therapeutic avenues. The latest WHO classification’s fifth edition underscores the pivotal role of genetic alterations in shaping patient outcomes, highlighting the need for personalized treatment strategies in the ongoing battle against this formidable disease [1].

Previously, GBM classification relied primarily on histological characteristics. However, advancements in molecular biology have unveiled a more intricate scenario. We now understand that GBM’s genetic profile involves a dynamic interplay of mutations, epigenetic factors, and disruptions in cellular signaling pathways. This molecular perspective has revolutionized our approach to understanding GBM’s biology, offering deeper insights into its complex pathophysiology [2]. The Cancer Genome Atlas (TCGA) project’s comprehensive genomic analysis of GBM marked a significant milestone in understanding the disease. This project identified key genetic alterations, including mutations in genes such as *TP53*, *PTEN*, *EGFR*, and *NF1*. These findings have been instrumental in elucidating the molecular mechanisms underpinning GBM, paving the way for targeted therapies and personalized medicine approaches in treating this challenging brain tumor [3].

While the majority of research has focused on GBM in Western populations, there’s an increasing emphasis on understanding its genetic profile in non-Western groups. For instance, a notable study on the Chinese population revealed unique genetic characteristics. This research uncovered a comparatively lower incidence of *EGFR* amplifications and a higher frequency of *TP53* mutations than typically observed in Western populations. This highlights the importance of exploring ethnic and regional variations in GBM genetics, which could lead to more tailored and effective treatment strategies globally [4]. These findings underscore the importance of considering ethnic and regional genetic variations in GBM. Such diversity can significantly influence diagnostic accuracy, prognostic assessments, and the effectiveness of therapeutic interventions. This perspective encourages a more personalized approach to managing GBM, taking into account the unique genetic makeup of diverse populations.

The conventional approach to treating GBM typically involves a sequence of surgical resection, followed by radiotherapy and chemotherapy using temozolomide (TMZ). Despite this regimen, the prognosis for GBM patients remains challenging, with median survival rates hovering around just 15 months. This reality underscores the urgency for more effective therapeutic strategies in the battle against this aggressive brain tumor [5]. The discovery of *MGMT* promoter methylation has become a key factor in predicting the efficacy of TMZ treatment in GBM patients. Research indicates that tumors with methylated *MGMT* promoters tend to respond more favorably to TMZ, leading to improved survival outcomes. This insight has been pivotal in guiding personalized treatment plans and enhancing the therapeutic approach for GBM patients [6]. Explorations into targeted therapies for GBM, particularly *EGFR* inhibitors for cases with *EGFR* amplification, have been conducted. However, the effectiveness of these treatments is often limited due to the tumor’s inherent heterogeneity and its capacity to develop resistance mechanisms. This complexity poses a significant challenge in achieving consistent success with targeted therapeutic strategies in GBM treatment [7]. In summary, current research underscores the genetic intricacies of GBM and the associated treatment challenges. While there has been notable advancement in understanding GBM’s molecular underpinnings, the journey from these insights to effective, practical treatments remains a significant and complex challenge. This highlights the ongoing need for innovative research and therapeutic strategies in the fight against this formidable brain cancer.

A retrospective study at Taichung Veterans General Hospital, spanning from 2010 to 2022, analyzed primary GBM patients and found that the average overall survival rate post-standard treatment was 18.7 months. This duration, slightly longer than the general average, provides valuable insights into patient outcomes within this specific population and timeframe [8]. This duration is significantly longer than the 14.6 months reported by Wang F. et al. [9] in 2018. Given the hypothesis that GBM patients from different ethnic backgrounds may exhibit distinct genetic profiles, we conducted a study involving 30 patients diagnosed with malignant brain tumors between 2009 and 2023. These tumors were classified as grade IV according to traditional WHO histopathological grading. These patients were divided into two groups based on a two-year survival threshold: those who survived for more than two years (Group A) and those who survived for less than two years (Group B). Using advanced Next-Generation Sequencing (NGS) molecular biology techniques, we performed gene sequencing and compared the results with the TCGA database. Our primary goal was to identify differences in genetic mutation sites between Eastern and Western populations. Furthermore, we aimed to identify factors that influence prognosis within these two patient groups.

## Material and methods

### Study population and tumor samples

Our study, a retrospective analysis at Taichung Veterans General Hospital in Taichung, Taiwan, evaluated 30 patients diagnosed with GBM between 2009 and 2022. Selection was based on the availability of comprehensive data and high-quality tumor samples. All patients underwent standardized therapy protocols, including surgical intervention, radiotherapy, and temozolomide-based chemotherapy, with bevacizumab for progressive GBM cases.

A limitation of our study is the absence of an initial power analysis, owing to its retrospective design. Our primary objective was to ensure a representative GBM patient sample, aiming to provide insightful findings despite this methodological constraint. This focus highlights the significance of a predetermined power analysis in future prospective studies to determine an adequate sample size that can reliably detect significant effects.

### Inclusion and exclusion criteria

Our inclusion criteria encompassed: (1) a confirmed diagnosis of GBM; (2) adherence to the standard therapy protocol; (3) availability of follow-up data and samples; (4) survival beyond one-month post-operation; and (5) average necrosis below 40% on top and bottom slides. The exclusion criteria were patients with incomplete follow-up data or inadequate sample quality for analysis. A validation cohort of 30 cases was carefully chosen from the primary group based on these criteria.

### The rationale for blinding or lack thereof

In our retrospective analysis, the nature of data collection and patient selection precluded the implementation of blinding. As a retrospective study, we relied on existing medical records and samples, which means the investigators were inherently aware of patient outcomes and treatment protocols during data analysis. However, to mitigate potential bias from this non-blinded approach, we employed strict inclusion and exclusion criteria and systematic data analysis methods. This methodology aimed to ensure the integrity and objectivity of our findings. Future studies, particularly prospective ones, should consider incorporating blinding techniques to further reduce bias and enhance the validity of results.

### Sample processing and data analysis

Post-surgery, tumor samples were immediately frozen for subsequent analysis. Overall survival (OS) was calculated from the surgery date to the date of death or the last follow-up examination.

### Ethical compliance

The Medical Ethics Committee of Taichung Veterans General Hospital approved our study protocols (Approval number: CF17263B-4), with the study concluding on March 1, 2023. All specimens were collected in compliance with institutional review board-approved protocols and anonymized to maintain patient confidentiality. Informed written consent was obtained from all participants, adhering to the principles of the Declaration of Helsinki. For a detailed overview of patient characteristics, please refer to Table 1.

**Table 1:** The study examined the relationships between overall survival over a 2-year period and survival under 2 years, as well as various patient characteristics.

	Patients (*n* = 30)	Follow ≤2 (*n* = 13)	Follow >2 (*n* = 17)	*p* value
	n %	n %	n %	
**Age, years**	53.0 (39.5–60.0)	50.0 (37.5–61.5)	54.0 (41.0–60.0)	0.542
**Age**				1.000
≤60	24 (80.0%)	10 (76.9%)	14 (82.4%)	
>60	6 (20.0%)	3 (23.1%)	3 (17.6%)	
**Gender**				0.880
Male	18 (60.0%)	8 (61.5%)	10 (58.8%)	
Female	12 (40.0%)	5 (38.5%)	7 (41.2%)	
**Tumor number**				0.698
Solitary	22 (73.3%)	9 (69.2%)	13 (76.5%)	
Multiple	8 (26.7%)	4 (30.8%)	4 (23.5%)	
**Tumor size**				0.666
>3 cm	7 (23.3%)	4 (30.8%)	3 (17.6%)	
≦3 cm	23 (76.7%)	9 (69.2%)	14 (82.4%)	
**Tumor occurrence**				0.290
Primary	26 (86.7%)	10 (76.9%)	16 (94.1%)	
Recurrence	4 (13.3%)	3 (23.1%)	1 (5.9%)	
**Bevacizumab** ^a^				0.030*
No used	14 (46.7%)	9 (69.2%)	5 (29.4%)	
Used	16 (53.3%)	4 (30.8%)	12 (70.6%)	
**DM**	1 (3.3%)	0 (0.0%)	1 (5.9%)	1.000
**HTN**	6 (20.0%)	2 (15.4%)	4 (23.5%)	0.672
**Follow month** ^a^	27.9 (12.6–55.8)	12.2 (7.0–15.2)	47.4 (33.9–94.4)	<0.001**

Fisher’s exact test was used for categorical data. For continuous data, the Mann-Whitney U test was employed, with results expressed as median (min-max) values. Statistical significance levels were set at *p < 0.05 and **p < 0.01.

^a^All patients received standard treatment, including TMZ and CCRT therapy. BEV was introduced only in cases of disease progression. *DM* Diabetes Mellitus, *HTN* Hypertension.

### DNA extraction and quality control

DNA was extracted from frozen samples using the QIAamp DNA Mini Kit (Qiagen, Hilden, Germany). The quantity and purity of the genomic DNA (gDNA) were assessed using the Qubit® 2.0 Fluorometer (Invitrogen, Carlsbad, CA, USA) and NanoDrop ND-1000 (Thermo Scientific, Wilmington, DE, USA). The fragmentation status of the gDNA was evaluated by the Agilent 2200 TapeStation system using the Genomic DNA ScreenTape assay (Agilent Technologies, Santa Clara, CA, USA), which generates a DNA Integrity Number (DIN). Additional quality control (QC) steps were performed to assess gDNA integrity using a multiplex Polymerase Chain Reaction (PCR) approach. In this approach, 30 ng of gDNA were amplified using three different-sized sets of primers targeting the Glyceraldehyde-3-Phosphate Dehydrogenase (GAPDH) gene (200-300-400 base pair), and the concentration of PCR products was determined using the Agilent 2100 Bioanalyzer instrument (Agilent Technologies). To estimate gDNA fragmentation, an Average Yield Ratio (AYR) value was calculated by comparing the yield ratio of each amplicon with a reference DNA.

### Targeted sequencing:

Each subject’s GBM tumor sample was collected for genomic DNA extraction. Genomic DNA was extracted from leukocytes using the QIAamp DNA Blood Mini Kit (Qiagen, Hilden, Germany) for subsequent next-generation sequencing analysis. Targeted sequencing was employed to sequence the specific regions of interest associated with GBM, including the complete exons of the *IDH1*, *TP53*, and *TERT* genes. Custom-designed probes and primers were used for these genes. The targeted panel used was the Carcinogens Gene Test Assay, utilized in clinical genetic trials at the Precision Medicine Laboratory of Taichung Veterans General Hospital.

Polymerase chain reaction (PCR) was performed to amplify and sequence the targeted DNA fragments. Library construction was carried out using the Qiagen Target Panel Kit (Qiagen, CDHS-15658z-227, Hilden, Germany), followed by quantification. The prepared library was loaded onto the Illumina Sequencing System (iSeq 100/MiniSeq, San Diego, CA, USA). FastQ files generated from the targeted DNA libraries were stored in CLC Genomics Workbench 12 (QIAGEN, Denmark), and variant calling was performed using QIAGEN Panel analyses. The pathogenicity assessment of variants was conducted using the Illumina Basespace Variant Interpreter. The pathogenic or likely pathogenic variants were further confirmed using the ClinVar database, a public archive providing information on human genomic variants and their associations with diseases, supported by clinical or functional evidence.

DNA libraries were generated using the QIAseq Human Comprehensive Cancer Panel, covering 275 genes (0.8 Mbp). Each sample utilized 40 ng of tumor genomic DNA following the manufacturer’s instructions. The prepared library underwent paired-end sequencing on the NovaSeq 6000 sequencer. DNA reads were aligned to the human reference genome GRCh37, and variant calling was performed using the integrated workflow in CLC Genomics Workbench 21. Somatic mutation annotation filtering and characterization were performed via QIAGEN Clinical Insight (QCI). Variants with a frequency below 3% were excluded, and pathogenic and likely pathogenic variants were identified based on the ACMG variant interpretation guidelines.

### Statistical analysis

Our analysis encompassed demographic data, which were presented as frequencies for categorical variables and examined using the chi-squared test or Fisher’s exact test, as appropriate. We assessed Overall Survival (OS) employing the Kaplan-Meier method, complemented by the log-rank test to discern survival differences. In our Cox proportional hazards regression analysis, we meticulously adjusted for key variables, including age, sex, and bevacizumab treatment. Crucially, we implemented Levene’s Test to evaluate the homogeneity of variance both within and across groups, thereby solidifying the validity of our statistical underpinnings. All statistical analyses were conducted using IBM SPSS, version 22.0. We considered *p* values less than 0.05 to be statistically significant. Moreover, the normal distribution of our data was confirmed through the Shapiro-Wilk test (*p* > 0.05), further bolstering the credibility of our results.

## Results

### Glioblastoma patient characteristics in the Taiwanese population

000These 30 patients were enrolled between February 2009 and September 2022, with ages spanning from 23 to 66 years, calculated from the date of surgery. The median age was 53 years. Within this cohort, Group A consisted of 17 patients who exhibited a survival of over two years, comprising 10 males and 7 females. Meanwhile, Group B comprised 13 patients who survived less than two years, consisting of 8 males and 5 females. The male-to-female ratio was 60% to 40%.

The maximum diameter and number of tumors were evaluated using preoperative Brain MRI scans with contrast. Furthermore, patients were stratified based on their received treatments, which encompassed TMZ + CCRT (Concurrent Chemoradiotherapy) and TMZ + CCRT+Bevacizumab. All treatments were in accordance with the current GBM treatment guidelines.

Furthermore, the impact of diabetes and hypertension on survival was evaluated at the time of diagnosis using Fisher’s exact test (Table 1). No statistically significant disparities were noted in terms of age, gender, tumor size, or number between the two groups. However, the utilization of Bevacizumab demonstrated a statistically significant correlation with prolonged survival (*p* = 0.030), indicating a positive association between Bevacizumab use and extended survival [8].

### The frequently mutated genes in glioblastoma within the Taiwanese population

**Fig. 1:**
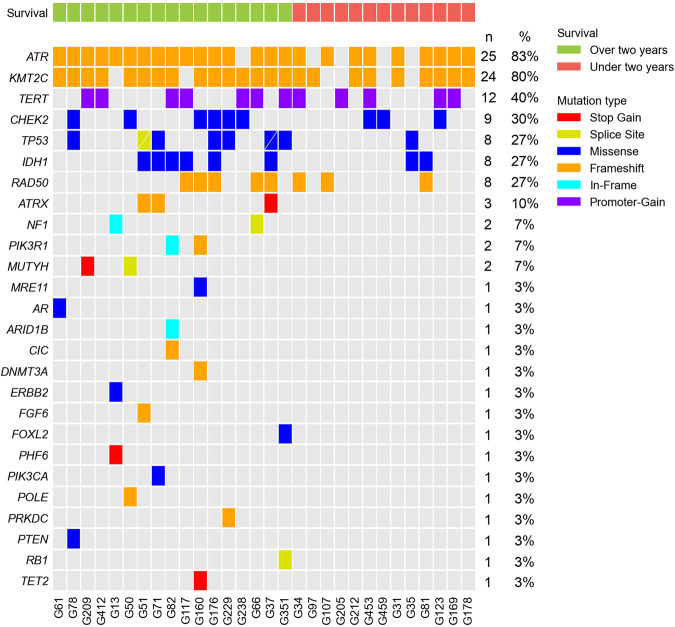
Genetic profiles and survival analysis in Taiwanese glioblastoma patients. This figure illustrates the genetic profiles of glioblastoma in the Taiwanese population, coupled with an investigation into the survival characteristics of patients. It provides a comprehensive analysis of genetic variations and their potential impact on patient survival outcomes, highlighting the significance of personalized medicine in the treatment of glioblastoma.

We employed NGS target panel techniques to analyze 30 glioblastoma samples. Utilizing the tertiary analysis system, QIAGEN Clinical Insight (QCI), we selected variants categorized as pathogenic or likely pathogenic, and filtered out those with a frequency of less than 3%. The samples were then stratified into two groups based on survival period. The results revealed a spectrum of mutations, encompassing missense mutations, nonsense mutations, frameshift mutations, and indels spanning the promoter, exon, and intron regions. Additionally, we conducted a quantification of the number of patients and the proportion of patients with each mutated gene. A visual representation of gene mutations was generated using Comut (Fig. 1) [10].

Upon closer examination of the heat map (Fig. 1), it is evident that the total mutation count in Group A patients surpasses that in Group B. We conducted Fisher’s exact test to scrutinize each mutated gene in both groups, excluding genes with zero mutations in both groups (such as *ATRX, MUTYH, PIK3R1*, etc.). Due to the limited sample size, our analysis results indicate that none of the mutations reached statistically significant levels (Table 2). However, there is a noteworthy trend towards a *p* value of 0.05 for *TP53*.

**Table 2:** Mutation Frequencies Determined by Sanger Sequencing.

Gene	Group A	Group B	Total count of patients with gene mutations.	*p v*alue
	(Total *N* = 17)	(Total *N* = 13)	(Total *N* = 30)	
ATR	16 (94.1%)	9 (69.2%)	25 (83.3%)	0.138
KMT2C	15 (88.2%)	9 (69.2%)	24 (80.0%)	0.360
TERT	7 (41.2%)	5 (38.5%)	12 (40.0%)	1.000
CHEK2	6 (35.3%)	3 (23.1%)	9 (30.0%)	0.691
TP53	7 (41.2%)	1 (7.7%)	8 (26.7%)	**0.092**
IDH1	6 (35.3%)	2 (15.4%)	8 (26.7%)	0.407
RAD50	5 (29.4%)	3 (23.1%)	8 (26.7%)	1.000
ATRX	3 (17.6%)	0 (0.0%)	3 (10.0%)	0.238
NF1	2 (11.8%)	0 (0.0%)	2 (6.7%)	0.492
PIK3R1	2 (11.8%)	0 (0.0%)	2 (6.7%)	0.492
MUTYH	2 (11.8%)	0 (0.0%)	2 (6.7%)	0.492
MRE11	1 (5.9%)	0 (0.0%)	1 (3.3%)	1.000
AR	1 (5.9%)	0 (0.0%)	1 (3.3%)	1.000
ARID1B	1 (5.9%)	0 (0.0%)	1 (3.3%)	1.000
CIC	1 (5.9%)	0 (0.0%)	1 (3.3%)	1.000
DNMT3A	1 (5.9%)	0 (0.0%)	1 (3.3%)	1.000
ERBB2	1 (5.9%)	0 (0.0%)	1 (3.3%)	1.000
FGF6	1 (5.9%)	0 (0.0%)	1 (3.3%)	1.000
FOXL2	1 (5.9%)	0 (0.0%)	1 (3.3%)	1.000
PHF6	1 (5.9%)	0 (0.0%)	1 (3.3%)	1.000
PIK3CA	1 (5.9%)	0 (0.0%)	1 (3.3%)	1.000
POLE	1 (5.9%)	0 (0.0%)	1 (3.3%)	1.000
PRKDC	1 (5.9%)	0 (0.0%)	1 (3.3%)	1.000
PTEN	1 (5.9%)	0 (0.0%)	1 (3.3%)	1.000
RB1	1 (5.9%)	0 (0.0%)	1 (3.3%)	1.000
TET2	1 (5.9%)	0 (0.0%)	1 (3.3%)	1.000

N, number. The statistical analysis employed Fisher’s exact test.

During the course of this experiment, we observed a higher prevalence of *TP53* mutations in Group A. This observation contradicts current Western research, which suggests an association between *TP53* mutations and a worsened prognosis in GBM [11].

### Characteristics of glioblastoma-associated variants in the genes *CHEK2*, *IDH1, TP53*, and *TERT* promoter in the Taiwanese population

**Fig. 2:**
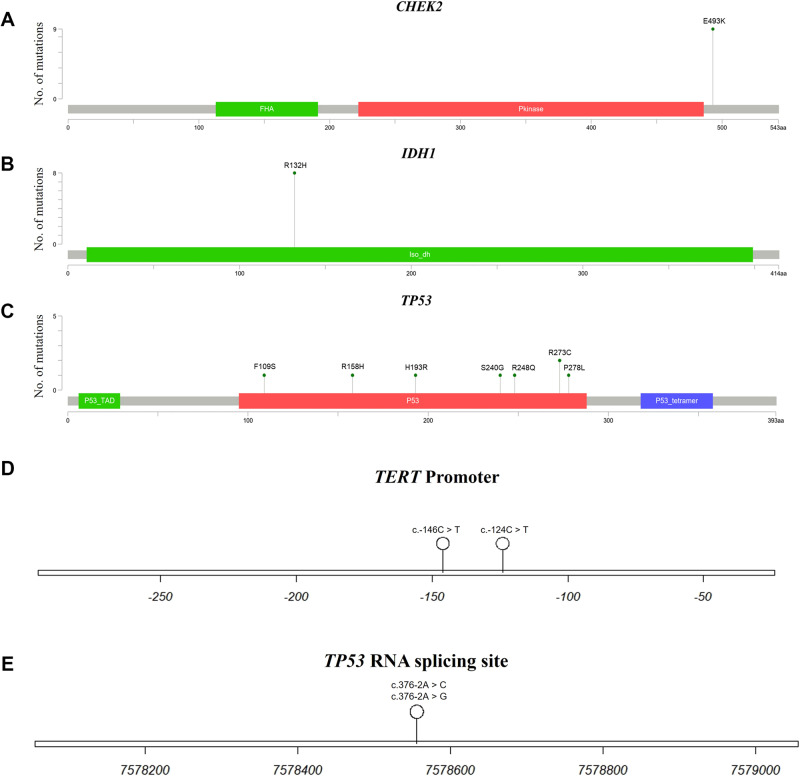
The lollipop plot. This figure illustrates amino acid substitutions in the **A** CHEK2, **B** IDH1, and **C** TP53 genes. The gray bar denotes the location of amino acids (aa). The circular lollipop marker indicates the specific site of amino acid substitution, with the height representing the variant count at those positions. Colored boxes represent distinct functional domains. **D** Schematic diagram of the TERT promoter. The bar marks the upstream regulatory region. The circular lollipop marker shows the specific site of nucleotide substitution. **E** Schematic diagram of the TP53 RNA splicing site. The bar denotes the TP53 gene in the chromosome 17 region using the GRCh37 reference genome. The circular lollipop marker indicates the specific site of nucleotide substitution.

We utilized lollipop plots to visually represent glioblastoma-associated gene mutation sites specific to the Taiwanese population. These mutation sites were categorized into coding and non-coding regions. Within the coding region, a single mutation in the *CHEK2* gene was identified as (c.1477 G > A, p.E493K) (Fig. 2A). In the *IDH1* gene, a solitary mutation was identified as (c.395 G > A, p.R132H) (Fig. 2B). In the *TP53* gene, seven mutations were identified as (c.326 T > C, p.F109S), (c.473 G > A, p.R158H), (c.578 A > G, p.H193R), (c.718 A > G, p.S240G), (c.743 G > A, p.R248Q), (c.817 C > T, p.R273C), and (c.833 C > T, p.P278L) (Fig. 2C). The coding region diagrams were generated using Mutation Mapper [12].

In the non-coding region, two variants were detected in the *TERT* promoter regulatory region: (c.-146C > T) and (c.-124C > T) (Fig. 2D). In the *TP53* gene, two variants were identified at the RNA splicing site: (c.376-2 A > C) and (c.376-2 A > G) (Fig. 2E). The non-coding region diagrams were prepared using track Viewer [13].

### The disparities in glioblastoma patient gene mutations between the Taiwanese and Western populations

This study endeavors to elucidate the distinctions in the genetic mutation profiles of glioblastoma between the Taiwanese population and Western populations. We curated data from the TCGA database and various publications to pinpoint the top 10 mutated genes in glioblastoma. Our observations indicate that mutations in the *ATR, KMT2C*, *TERT, RAD50*, and *CHEK2* genes are more prevalent in the Taiwanese population, whereas the mutation frequencies of *TP53, IDH1, ATRX, NF1*, and *PIK3R1* are more akin to those in Western populations (Table 3). Additional details regarding these six TCGA projects can be found in the Supplementary Materials.

**Table 3:** The data presented in this table has been sourced from the public records of cBioPortal, with a specific focus on CNS/brain studies, particularly targeting glioblastoma.

Selected patients/samples	1. Glioblastoma multiforme (TCGA, PanCancer Atlas)	2. Glioblastoma Multiforme (TCGA Firehose Legacy)	3. Glioblastoma (TCGA, Nature 2008)	4. Glioblastoma (TCGA, Cell 2013)	5. Glioblastoma Columbia, Nat Med, 2019)	6. Glioblastoma (CPTAC, Cell 2021)	7. Glioblastoma (Veteral General hospital Taichung, 2023)
	585/592	606/619	206/206	577/577	42/42	99/99	30/30
Mutated gene and frequency (%)							
1	PTEN (33.5%)	PTEN (31.0%)	TP53 (34.1%)	PTEN (23.7%)	PTEN (18.8%)	TP53 (32.3%)	ATR (83%)
2	TP53 (31.5%)	TP53 (29.00%)	PTEN (31.9%)	EGFR (21.0%)	IDH1 (12.5%)	PTEN (27.3%)	KMT2C (80%)
3	TTN (25.4%)	EGFR (26.6%)	EGFR (16.5%)	TP53 (20.3%)	RYR2 (12.5%)	TTN (20.2%)	TERT (40%)
4	EGFR (23.7%)	TTN (25.5%)	NF1 (14.3%)	TTN (19.9%)	NF1 (9.4%)	MUC4 (18.2%)	CHEK2 (30%)
5	MUC16 (15.4%)	MUC (16.2%)	PIK3R1 (9.9%)	MUC16 (12.4%)	MUC16 (9.4%)	EGFR (17.2%)	TP53 (27%)
6	FLG (13.4%)	PIK3R1 (11.4%)	RB1 (9.9%)	PIK3R1 (11.0%)	PIK3CA (9.4%)	NF1 (15.2%)	IDH1 (27%)
7	NF1 (11.6%)	FLG (11.0%)	ERBB2 (7.7%)	NF1 (9.3%)	DSG3 (9.4%)	PIK3CA (11.1%)	RAD50 (27%)
8	RYR2 (10.8%)	PIK3CA (11.0%)	PIK3CA (6.6%)	PIK3CA (8.9%)	FAM83H (9.4%)	ATRX (10.1%)	ATRX (10%)
9	PIK3R1 (9.8%)	NF1 (11.0%)	MSH6 (4.4%)	SPTA1 (8.9%)	TP53 (9.4%)	RB1 (10.1%)	NF1 (7%)
10	PIK3CA (9.6%)	RYR2 (10.0%)		FLG (8.2%)	MYH1 (6.3%)	RYR2 (9.1%)	PIK3R1 (7%)
**Source of Data**	USA	USA	USA	USA	USA	USA	Taiwan

We have curated six studies that conducted comprehensive whole-genome analyses. The selected studies are as follows:

Glioblastoma Multiforme TCGA PanCancer data.

TCGA Glioblastoma Multiforme. Source data obtained from GDAC Firehose, formerly referred to as TCGA Provisional.

Targeted sequencing of 91 out of 206 primary glioblastoma tumors (143 with matched normals) from the Cancer Genome Atlas (TCGA) Glioblastoma Project.

Whole-exome and/or whole-genome sequencing of 291 out of 577 glioblastoma tumor/normal pairs from the Cancer Genome Atlas (TCGA) Glioblastoma Project.

Whole-exome sequencing of 32 out of 42 glioblastoma patients with matched normals.

Proteogenomic and metabolomic characterization of human glioblastoma. Whole genome or whole exome sequencing of 99 samples, as produced by CPTAC.

Furthermore, data generated by our institution is provided for comparative analysis. This table enumerates the top 10 mutated genes for each of the studies.

### Statistical analysis of the effects of *IDH1* and *TP53* mutations on survival rate and age distribution of patients

**Fig. 3:**
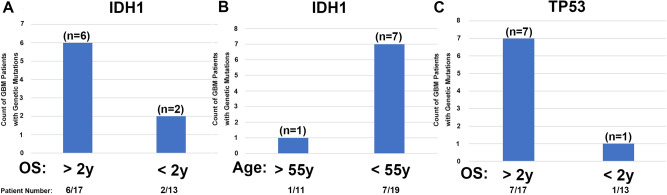
The Bar Chart Statistical Analysis illustrates the Frequency of Genetic Mutations in GBM Patients. **A** Count of patients with IDH1 mutation in the two-year survival rate. **B** Count of patients with IDH1 mutation aged 55 years and below. **C** Count of patients with TP53 mutation in the two-year survival rate. OS: overall survival.

Following the NGS results, we conducted a thorough statistical analysis. Due to the limited sample size, none of the mutations yielded statistically significant results. However, we identified the genes *IDH1* and *TP53* as having potential statistical significance. Regarding *IDH1*, we observed six patients in Group A and two patients in Group B (Fig. 3A). The age distribution analysis for *IDH1* mutations indicated one patient below the age of 55 and seven patients aged 55 or above (Fig. 3B). These findings suggest that mutations in *IDH1* among glioblastoma patients in Taiwan are linked with a more favorable prognosis, and the majority of patients with *IDH1* mutations are under the age of 55.

As for *TP53*, our investigation revealed seven patients in Group A and one patient in Group B (Fig. 3C). This outcome suggests that mutations in *TP53* among glioblastoma patients in Taiwan are associated with a better prognosis.

Based on our NGS analysis data, Table 3 illustrates the heat map. *ATR* stands out as the most frequently mutated gene, accounting for 83% of all mutations. Following *KMT2C, TERT*, and *CHEK2* mutations are the next most prevalent. For further specifics, please refer to the gene mutation heat map and Table 3.

In our study analyzing the top 10 genetic mutations in the Taiwanese population, we utilized a Cox proportional hazards regression model. This model was adjusted for age, gender, and bevacizumab treatment, as detailed in Table 4. Our findings indicate a notable association between the *IDH1* mutation and patient prognosis. Specifically, compared to the wild type, the *IDH1* mutation shows a hazard ratio (HR) of 0.31 (95% CI: 0.11–0.83) with a *p* value of 0.020 in the multivariate analysis. This suggests a significantly lower risk ratio (*p* < 0.05), indicating that glioblastoma patients with the *IDH1* mutation may have a higher survival rate. Our study’s findings reveal that patients harboring *IDH1* mutations demonstrate a notably prolonged survival rate. According to the World Health Organization’s 2021 glioma classification protocol, which emphasizes the significance of *IDH1* mutations in its hierarchical categorization and integrates histopathological features, including microvascular proliferation and/or necrosis, these patients are classified as ‘Astrocytoma, *IDH*-mutant, CNS WHO Grade 4’ [14]. This classification demonstrates a comparatively favorable prognosis within the CNS WHO Grade 4 spectrum, aligning with our study’s results. This correlation echoes the trends observed in recent literature [14, 15]. In contrast, the *TP53* mutation did not demonstrate statistical significance in the risk ratio. After adjustments, the HR for the mutation group was 0.49 (95% CI: 0.21–1.17), with a *p* value of 0.107. Despite the decreased HR value, the lack of statistical significance underscores the need for further investigation with larger sample sizes. Other genetic mutations analyzed did not show significant effects in this multivariate analysis.

**Table 4:** Comparative analysis of hazard ratios for specified gene mutations in univariate and multivariate models.

	No. of subjects	No. of cases (%)	Univariate			Multivariable^a^		
			HR	95%CI	*p* value	HR	95%CI	*p* value
ATR								
Wild type	5	4 (14.8%)	Reference			Reference		
Mutation	25	23 (85.2%)	0.61	(0.20–1.82)	0.374	0.53	(0.17–1.66)	0.278
KMT2C								
Wild type	6	6 (22.2%)	Reference			Reference		
Mutation	24	21 (77.8%)	0.37	(0.14–0.99)	0.049*	0.58	(0.21–1.66)	0.313
TERT								
Wild type	18	17 (63.0%)	Reference			Reference		
Mutation	12	10 (37.0%)	0.98	(0.44–2.16)	0.956	0.95	(0.37–2.43)	0.920
CHEK2								
Wild type	21	20 (74.1%)	Reference			Reference		
Mutation	9	7 (25.9%)	0.48	(0.20–1.16)	0.103	0.81	(0.28–2.31)	0.690
TP53								
Wild type	22	19 (70.4%)	Reference			Reference		
Mutation	8	8 (29.6%)	0.72	(0.31–1.67)	0.444	0.47	(0.18–1.18)	0.106
IDH1								
Wild type	22	19 (70.4%)	Reference			Reference		
Mutation	8	8 (29.6%)	0.63	(0.26–1.50)	0.294	0.31	(0.11–0.83)	0.020*
RAD50								
Wild type	22	19 (70.4%)	Reference			Reference		
Mutation	8	8 (29.6%)	1.10	(0.47–2.55)	0.830	0.58	(0.22–1.51)	0.264
ATRX								
Wild type	27	24 (88.9%)	Reference			Reference		
Mutation	3	3 (11.1%)	0.63	(0.19–2.15)	0.464	0.25	(0.06–1.00)	0.050
NF1								
Wild type	28	25 (92.6%)	Reference			Reference		
Mutation	2	2 (7.4%)	1.54	(0.35–6.82)	0.567	0.76	(0.16–3.56)	0.731
PIK3R1								
Wild type	28	25 (92.6%)	Reference			Reference		
Mutation	2	2 (7.4%)	0.41	(0.09–1.80)	0.238	0.74	(0.14–3.83)	0.723

Cox proportional hazard regression. *p < 0.05, **p < 0.01. Bold indicates a statistically significant difference with a p-value less than 0.05.

^a^Adjustments made for age, sex, and bevacizumab treatment. HR denotes Hazard Ratio.

## Discussion

### *EGFR* mutations in the Taiwanese population in our study

The data from the TCGA database, accessed through cBioPortal and outlined in Table 3 [16–19] shows that prevalent mutations, especially in *PTEN* and *EGFR*, are observed less frequently in our patient cohort compared to others. In particular, *EGFR* mutations are completely absent, as demonstrated in the mutation heat maps for both Group A and Group B. To corroborate these observations, we employed first-generation SANGER sequencing and verified the nonexistence of *EGFR* mutations in all 30 patients in our study. This contrasts with the findings in Asian populations; for instance, Fukushima and Favereaux [16] observed a 2% mutation rate in *EGFR* kinase regions of Japanese glioblastomas using SSCP and DNA sequencing. This rate is markedly different from the polymorphic allele frequency in Swiss glioblastomas, which show a lower mutation rate in the Japanese group. Our results are consistent with these findings, indicating a similar trend in mutation rates across different populations.

In the classic subtype of The Cancer Genome Atlas (TCGA), a high frequency of *EGFR* amplification is typically noted [20]. However, this trend is not commonly seen in Chinese patients [4]. Similarly, Nayuta HIGA et al. [21], utilizing NGS, reported a lower rate of *EGFR* amplification in GBM in Asian patients compared to those in other regions. Our research confirms a low frequency of EGFR gene mutations in both Groups A and B of our study. It is important to note that our study did not evaluate the extent of *EGFR* amplifications. A more focused investigation into *EGFR* amplifications could reveal significant differences in the oncogenic pathways and treatment approaches for GBM between Eastern and Western populations. This differentiation could be crucial for developing region-specific treatment strategies.

### *TP53* mutation analysis

In our study, both Group A and Group B exhibit a p-value close to 0.05 in association with *TP53* mutations, hinting at a potential link to enhanced survival rates. This observation aligns with the findings of Noor H, Briggs NE, et al. [22], who reported that *TP53* mutations significantly improved overall survival in astrocytoma patients. Their research highlighted the presence of hotspot mutations in *TP53*, particularly at codon 273, in 33% (17 out of 51) of astrocytoma samples. Retrospective analysis indicated markedly better clinical outcomes in patients who received chemotherapy, suggesting that specific mutations in *TP53*, especially at codon 273, could be critical in determining the effectiveness of therapy in astrocytomas and, consequently, in affecting survival rates. In our study, we also detected mutations at codon 273 (specifically R273C (c.817 C > T)) in two patients, labeled G71 and G35. However, these patients were part of Groups A and B, respectively, and thus, we could not establish statistical significance for this observation.

The research by Lauren R. Olafson et al. [23] revealed that the *TP53* gene in G53 tumors (classified as secondary GBM) harbors a c.818 G > A (p.R273H) mutation at codon 273 of exon 8. This particular mutation leads to a gain of function (GOF) in the p53 protein, which could be responsible for increased tumor aggression, proliferation, invasiveness, and metastatic potential. Given these findings, it becomes crucial to further investigate how missense mutations at various locations within the *TP53* gene contribute to functional changes and influence tumor progression. Additionally, identifying any differences in mutation sites between Eastern and Western populations calls for a more comprehensive dataset. This would help in understanding regional variations in tumor genetics and could guide targeted treatment strategies.

### Bevacizumab treatment in GBM recurrence

In a study conducted by our institution focusing on the use of bevacizumab in recurrent GBM, we employed the Polymerase Chain Reaction (PCR) - Restriction Fragment Length Polymorphism (RFLP) technique to investigate the *CDKN1A* (*p21*) c.93 C > A gene polymorphism [8]. This research included 139 glioblastoma patients and explored the prevalence of different CDKN1A c.93 C > A genotypes. Although we found no direct link between these genotypes and the overall survival rate in glioblastoma patients, our findings did reveal a notable survival benefit in patients possessing certain genotypes (Arg/Arg and Arg/Ser). This advantage was observed in those who received a combination of concurrent chemoradiotherapy and bevacizumab monoclonal antibody treatment, as opposed to those treated with chemoradiotherapy alone. Additionally, our study suggests a positive association between bevacizumab use and prolonged survival in these patients.

In Japan, significant research has been conducted on the use of bevacizumab in GBM patients. These studies conclude that bevacizumab monoclonal antibody treatment can be particularly beneficial for tumors in patients who lack *MGMT* methylation [24]. Additionally, a post-market surveillance study in Japan examined the safety and effectiveness of bevacizumab for treating malignant gliomas [24]. While this study did not pinpoint specific genetic variations that might predict a positive response to bevacizumab, it did confirm that the use of bevacizumab in GBM patients is associated with extended survival. Furthermore, it underscored that bevacizumab is a safe and effective treatment option for these patients.

## Conclusion

In Taiwan’s GBM patient cohort, bevacizumab use correlates positively with increased survival rates, underscoring its safety and effectiveness. Notably, the prevalence of *EGFR* mutations in this group is lower than in Western counterparts, hinting at distinct carcinogenesis pathways. This calls for further detailed research on *EGFR* mutations and amplifications to deepen our understanding and improve treatment strategies. Additionally, our study links *TP53* mutations to better survival outcomes, although the specific impacts of missense mutations require further investigation. This pioneering study utilizing NGS technology sheds new light on the genetic variations in Taiwanese GBM patients, significantly enriching our knowledge of their genetic profile.

### Supplementary information

The six TCGA database projects

## Data Availability

Our research data, housed at Taichung Veterans General Hospital, is subject to specific licensing restrictions that limit its public availability. This data was primarily utilized for our study. Requests for data access can be made to the corresponding authors and are subject to hospital approval. For detailed insights into our Next-Generation Sequencing (NGS) methods and results, please refer to https://www.ncbi.nlm.nih.gov/sra/. Note: The accession number mentioned must match the raw data provided in the Supplementary Materials. In our commitment to rigorous data-sharing standards, we are implementing the following measures: Accession Codes: We will obtain and publish unique accession codes for each dataset from established public repositories. Regulatory Compliance: We are committed to adhering to all relevant licensing, ethical standards, and confidentiality agreements. In cases where public data sharing is restricted, we will outline alternative access methods. Collaborative Compliance: Our team will collaborate closely with co-authors and affiliated institutions to secure all necessary approvals and ethical permissions for data distribution.
