# Supplementary material for: Genetic mutation patterns among glioblastoma patients in the Taiwanese population – insights from a single institution retrospective study
Source: Cancer Gene Ther. 2024 Feb 28;31(6):894–903. doi: 10.1038/s41417-024-00746-y (PMC11192627; doi:10.1038/s41417-024-00746-y)
Supplement: Supplementary file 1 — The six TCGA database projects [file 41417_2024_746_MOESM1_ESM.doc]

**Additional Information:**

For the six TCGA database projects we referenced (Table 3), the pertinent links and references are provided below:

Project 1: Glioblastoma Multiforme (TCGA, PanCancer Atlas). Available at: <https://datacatalog.mskcc.org/dataset/10414> Retrieved on September 3, 2023.

Project 2: Glioblastoma Multiforme (TCGA, Firehose Legacy). Available at: <https://datacatalog.mskcc.org/dataset/10472> Retrieved on September 3, 2023.

Project 3: Glioblastoma (TCGA, Nature 2008)[1].

Project 4: Glioblastoma (TCGA, Cell 2013)[2].

Project 5: Glioblastoma (Columbia, Nat Med. 2019)[3].

Project 6: Glioblastoma (CPTAC, Cell 2021)[4].

**References:**

1. Cancer Genome Atlas Research N: **Comprehensive genomic characterization defines human glioblastoma genes and core pathways**. *Nature* 2008, **455**(7216):1061-1068.

2. Brennan CW, Verhaak RG, McKenna A, Campos B, Noushmehr H, Salama SR, Zheng S, Chakravarty D, Sanborn JZ, Berman SH *et al*: **The somatic genomic landscape of glioblastoma**. *Cell* 2013, **155**(2):462-477.

3. Zhao J, Chen AX, Gartrell RD, Silverman AM, Aparicio L, Chu T, Bordbar D, Shan D, Samanamud J, Mahajan A *et al*: **Immune and genomic correlates of response to anti-PD-1 immunotherapy in glioblastoma**. *Nat Med* 2019, **25**(3):462-469.

4. Wang LB, Karpova A, Gritsenko MA, Kyle JE, Cao S, Li Y, Rykunov D, Colaprico A, Rothstein JH, Hong R *et al*: **Proteogenomic and metabolomic characterization of human glioblastoma**. *Cancer Cell* 2021, **39**(4):509-528 e520.
